# Supplementary material for: The interactome of intact mitochondria by cross-linking mass spectrometry provides evidence for coexisting respiratory supercomplexes
Source: Mol Cell Proteomics. 2017 Dec 8;17(2):216–32. doi: 10.1074/mcp.RA117.000470 (PMC5795388; doi:10.1074/mcp.RA117.000470)
Supplement: Supplemental Data [file supp_RA117.000470_133922_0_supp_23978_fzffwf.zip › spectra_annotation/mito_spectra_annotation/138-2-16-1-9-1.pdf]

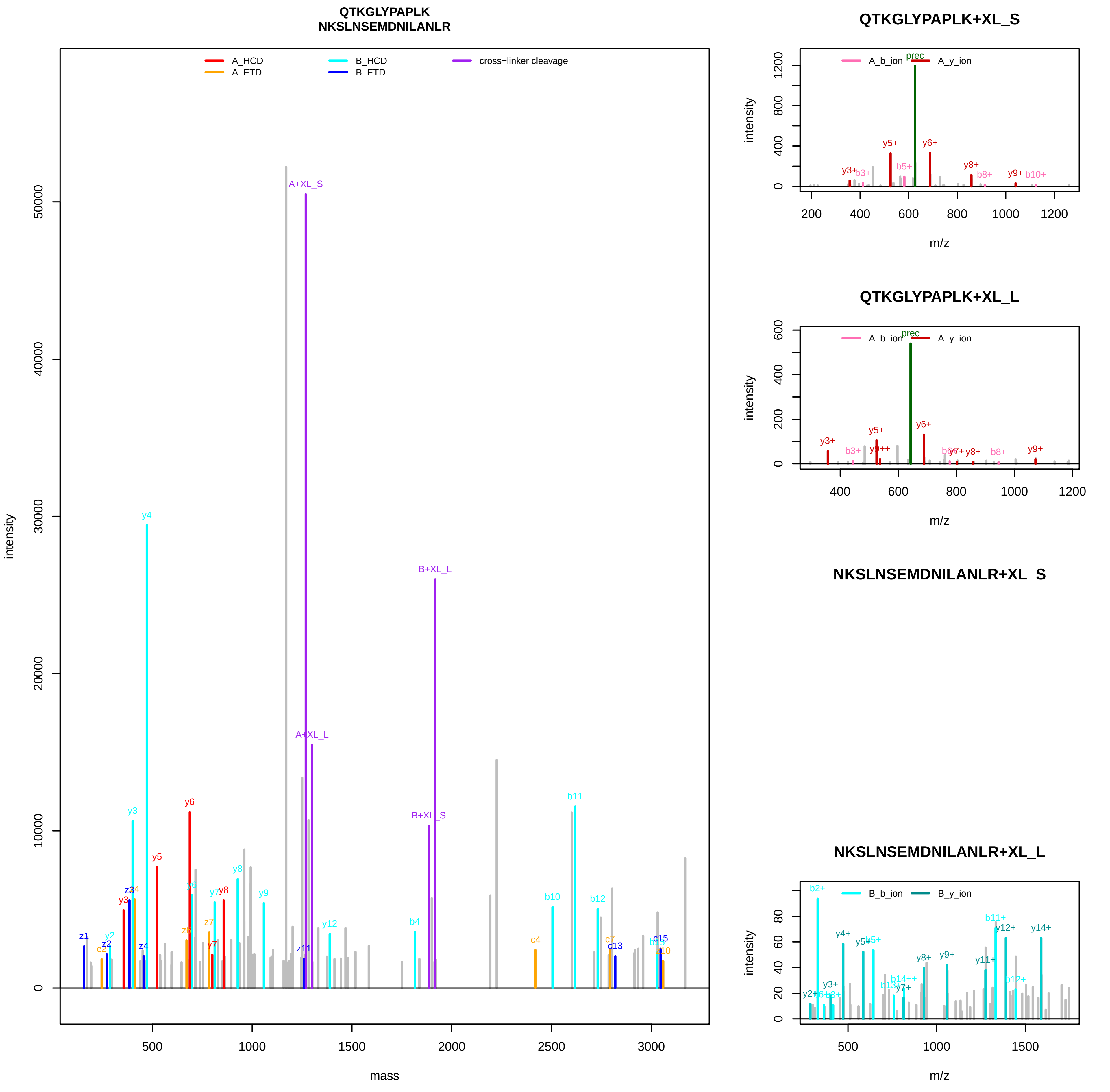
QTKGLYPAPLK
NKSLNSEMDNILANLR
A_HCD
A_ETD
B_HCD
B_ETD
cross−linker cleavage
A+XL_S
A+XL_L
B+XL_L
B+XL_S
y4
y3
y5
y6
y7
y8
y9
y12
z1
z2
z3
z4
z6
z7
z11
c2
c4
c7
c13
c15
b4
b10
b11
b12
intensity
mass
0
10000
20000
30000
40000
50000
500
1000
1500
2000
2500
3000
QTKGLYPAPLK+XL_S
A_b_ion
A_y_ion
prec
y3+
b3+
y5+
b5+
y6+
y8+
b8+
y9+
b10+
intensity
m/z
QTKGLYPAPLK+XL_L
y3+
b3+
y5+
y6+
y7+
y8+
b8+
y9+
NKSLNSEMDNILANLR+XL_S
NKSLNSEMDNILANLR+XL_L
B_b_ion
B_y_ion
b2+
y2+
y3+
y4+
y5+
b5+
y7+
y8+
y9+
y11+
b11+
y12+
b12+
y14+
b14++
